# A Rebuttal of McKeague et al. (2024): “Conservation Detection Dogs: A Critical Review of Efficacy and Methodology”

**DOI:** 10.1002/ece3.72389

**Published:** 2025-10-27

**Authors:** Ngaio L. Richards

**Affiliations:** ^1^ Forensics & Field Specialist, Working Dogs for Conservation Missoula Montana USA

## Abstract

This letter to the editor is offered in rebuttal of “Conservation detection dogs: A critical review of efficacy and methodology,” by McKeague et al. (2024), published in Ecology and Evolution. Drawing from over a decade of experience as a conservation detection dog handler within one of the longest standing organizations in North America, and with over two decades as a field biologist and researcher, the author shares insights around fundamental misgivings and concerns regarding the methodology used for the selection and review of studies, the core analyses conducted, and the ensuing conclusions and recommendations. A detailed rebuttal/critical review has been prepared and is available via request from the author.

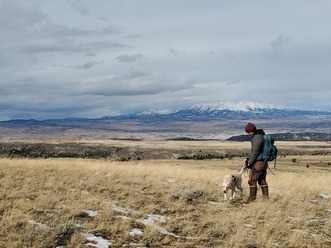

In February of 2024, Ecology and Evolution published “Conservation detection dogs: A critical review of efficacy and methodology,” by McKeague and colleagues. In my professional opinion, McKeague et al. (2024) neither sufficiently encapsulate nor accurately represent the current state of conservation detection to warrant its framing as a review paper. Furthermore, the underlying approach and narrative around “efficacy” and methodological standardization are often that of a problem in search of a solution, while overlooking evidence to the contrary within the paper's own references section. Fundamentally, the lens through which McKeague and co‐authors focused on “efficacy” and “standardization” frequently precluded consideration of effectiveness, usefulness, and viability.

The document I initially prepared as a letter to the editor developed of necessity into a detailed, point‐by‐point critical review/rebuttal of McKeague et al. (2024). I also reworked the paper's core analyses and tabulated data in an interactive spreadsheet in further support of my points. Here, I will summarize my concerns, concluding with my rationale for not jointly submitting the critical review/rebuttal to Ecology and Evolution for publication but rather making it available elsewhere.

My remarks draw from perspectives I have acquired over nearly 15 years as a conservation detection dog (CDD) handler, field biologist/ecologist, and researcher with Working Dogs for Conservation, based in Montana, USA. In this capacity, I have partnered directly with dogs of various breeds and pedigrees to search for a variety of conservation targets: fecal matter, aka “scat” (of San Joaquin kit fox, blunt‐nosed leopard lizard, black and brown bear, moose, Cross River gorilla, American mink, river otter), plants (e.g., dyer's woad, yellow star thistle), insect‐related targets (e.g., emerald ash borer beetle larvae and infested wood, bumble bee nest material), and conducted watercraft inspections for invasive zebra and quagga mussels. I have also initiated or helped develop applied canine detection initiatives including contaminants monitoring, collaborated on social justice initiatives, coordinated on survey and study design, and often liaise with our genetics laboratory partners. I have co‐authored and edited numerous conservation detection publications, including on the aforementioned targets. Finally, I have a firm footing in academia and academic research, as one of the coordinators of the Wildlife Forensic Sciences and Conservation program at the University of Florida and an instructor/mentor to the students. Like several of my WD4C colleagues, I frequently review papers on conservation detection, as well as on wider ecological monitoring initiatives, contaminant monitoring, veterinary and environmental forensics, and nontarget organism exposure to pesticides and veterinary products. The perspectives and arguments presented in this letter—and in the accompanying critical review—are my own and may not fully reflect the organization's official position. Nonetheless, I am grateful to have the encouragement of my colleagues in sharing them.

In the abstract, McKeague et al. (2024) state that:

We describe the efficacy of CDD across species and situational contexts like training and fieldwork…

CDD are generally quicker, can cover wider areas and find more samples than humans and other analytical tools. However, their efficacy varies between studies; methodological and procedural standardization in the field is lacking. Considering the cost of deploying a CDD team and the limited financial resources within conservation, it is vital that their performance is quantified and reliable…

It is clear that CDDs can be effective and applied to possibly limitless conservation scenarios, but moving forward researchers must provide more consistent and detailed methodologies so that comparisons can be conducted, results are more easily replicated and progress can be made in standardizing CDD work.

On the face of it, these statements may seem reasonable. However, the supporting information and arguments are not always framed accurately and are, in some cases, misleading. As such, my misgivings about this review paper fall into the following distinct categories:

1. The authors do not acknowledge intrinsic sources of variation in CDD “efficacy.” Arguably, the main source of variation and differing “efficacy” arises from the sheer breadth of conservation targets, and the different scent iterations (and scenting or detection challenges) that each may present. Examples include: live animals, carcasses, occupied burrows, and fecal deposits (scat); or: various life cycle stages of a plant such as early leafing (rosette) to flowering and seeding, and subterranean remnant roots.
2. The definition of “efficacy” and promotion of methodological standardization as highlighted both detract from certain core considerations. Crucially, methodological standardization does not equate to quality assurance, nor does it guarantee successful or viable operational outcomes.
3. The inclusion and exclusion criteria, as described in the Methods section (on page 3), were fundamentally flawed. These criteria resulted in omission of relevant studies and references, which in some cases would have contradicted the main thrust of the review or offered a differing perspective.
4. Rather than being collated and analyzed together (see table 2), reported parameters from controlled/laboratory studies—where variation is easier to control or manipulate—should have been separately considered from studies of a more operational nature. As one progresses from formative/controlled training to operational/real‐world settings, a drop in measured efficacy is not atypical. The authors seemed to suggest that equivalent levels of specificity, precision, or efficacy can or should be consistently achieved from training to fielding, across different species, targets, and projects, or even at times for the same species and targets—but that is not in alignment with empirical evidence or operational realities in this profession.
5. Field‐preparedness is conflated with field‐readiness. Field‐testing trials are often executed in a way that reflects whether a dog or dog‐handler team can find placed samples in situ with high/satisfactory precision and specificity. However, this is still not tantamount to field‐*readiness*, especially if sample placement is not consistent with how the target occurs in its natural setting. There can be a built‐in acclimation period at the deployment site, transitioning from training (i.e., can the dog and team recognize/find their target?) to operational (i.e., is that target in fact there for them to find?). Sometimes, the viability or efficacy of the application itself cannot be fully ascertained until the final stages—in real‐world settings—and that assessment might be the ultimate reason for conducting the work.
6. Sample sizes—whether numbers of dogs participating in a study or target samples found—are characterized as “small,” without due consideration of operational realities and successes, or of ecological and biological significance, rather than or alongside statistical significance. In tandem, neither replication nor replicability are always the desired objective; sometimes the aim is to establish the viability of an application then build upon it to maximize usefulness and effectiveness.
7. The authors focus on dogs and canine performance—with negligible consideration of the human component, or optimal pairing of dogs and handlers with varying degrees of experience to maximize learning, performance, and team cohesion. McKeague et al. (2024) do eventually allude to the importance of the dog‐handler relationship, stating that “CDDs must work as a team alongside a human handler who oversees searches, verifies finds and reinforces training. As such, the handler also plays a crucial role in CDD outcomes.” But this statement is provided almost as an afterthought, on page 12 of 14 (excluding the references section).
8. Legitimate constraints and limitations around reporting CDD parameters and publishing results are not considered by the authors. The 67 studies cited by McKeague et al. (2024) were published across 43 different journals/publication outlets having different scopes for reporting on CDD variables around the broader context of the work and findings. Also, not all work that has been successfully undertaken is found in the published literature, and broad outreach with colleagues and professional entities can round out understanding of previous and current efforts underway.
9. Certain studies have been cited without full context, or inaccurately—as I confirmed directly with the respective authors—to support assertions made by McKeague et al. (2024); certain inaccuracies are also present.

My rebuttal/critical review goes into considerably more detail on all these aspects. However, I do wish to highlight that I have firsthand knowledge of inaccurate and misleading referencing of some of the studies, either by virtue of having served as a canine handler and/or field lead on the project, and/or as a co‐author of the cited studies. Overall, I feel that the McKeague et al. (2024) paper performs a disservice to our profession by failing to convey crucial nuance of how—incorporating decades of lessons learned—we work and evolve with dogs, canine handlers, dog teams, and supporting practitioners on both novel and established targets. Moreover, the review appears to overlook a fundamental reality of this field—that some degree of uncertainty and variation is inherent, particularly at the operational level where practitioners must translate preparedness into true operational readiness.

Conservation detection is a rapidly growing field, and such swift expansion is not without valid concerns. As more people learn about this work and wish to embark upon it, there will be varying ranges of dedication and expertise—from people volunteering with their pets to people who have devoted their entire careers to this field. While interest, exploration, and entry into this work are encouraged, it is absolutely paramount that dogs, handlers, and teams be adequately vetted, trained, and deployed operationally to ensure no detriment to the profession. It is my opinion that the approach and conclusions of McKeague et al. (2024) impart misleading impressions—especially to those new to the discipline and having professional aspirations or as potential colleagues and collaborators.

Finally, my rebuttal/critical review and its supporting analyses, initially submitted to Ecology and Evolution as a letter to the editor, were too lengthy and detailed to serve that purpose. Although favorably reviewed, with the suggestion to submit separately to Ecology and Evolution, the associated publication fee is prohibitive. Publication credit is not my priority or aim, so the rebuttal/critical review and supporting materials will instead be publicly available through my ResearchGate page.

## Author Contributions

**Ngaio L. Richards:** conceptualization (lead), data curation (lead), formal analysis (lead), investigation (lead), methodology (lead), project administration (lead), resources (lead), visualization (lead), writing – original draft (lead), writing – review and editing (lead).

## Conflicts of Interest

The author declares no conflicts of interest.

## Data Availability

The sole data considered in this work is taken from that already available in the rebutted paper, McKeague et al. (2024).
